# The interplay between vitamin C and thyroid

**DOI:** 10.1002/edm2.432

**Published:** 2023-05-29

**Authors:** Bahareh Farasati Far, Amir Hossein Behnoush, Elina Ghondaghsaz, Mohammad Amin Habibi, Amirmohammad Khalaji

**Affiliations:** ^1^ Department of Chemistry Iran University of Science and Technology Tehran Iran; ^2^ School of Medicine Tehran University of Medical Sciences Tehran Iran; ^3^ Undergraduate Program in Neuroscience University of British Columbia Vancouver British Columbia Canada; ^4^ Gene, Cell & Tissue Research Institute Tehran University of Medical Science Tehran Iran; ^5^ Clinical Research Development Center Qom University of Medical Sciences Qom Iran

**Keywords:** ascorbic acid, hormone imbalance, thyroid cancer, thyroid function, thyroid hormones, vitamin C

## Abstract

**Introduction:**

Vitamin C (ascorbic acid) is a water‐soluble vitamin, that plays a key role in the prevention and treatment of scurvy. As vitamin C is an antioxidant and thyroid function may be affected and may affect vitamin C levels, for the first time, we aimed to provide a detailed review of all human studies evaluating the different roles of vitamin C in the thyroid gland. Thyroid cancers, goitre, Graves' disease and other causes of hyperthyroidism and hypothyroidism were the conditions discussed in this study. Furthermore, vitamin C addition to other medications such as levothyroxine was also reviewed.

**Methods:**

In this study, we reviewed the relevant literature regarding the association between vitamin C and thyroid diseases using original studies from PubMed, Scopus, Embase, and Web of Science.

**Results:**

In this review, we found anti‐cancer effects for intravenous (IV) administration of vitamin C in addition to the beneficial effects of using it in combination with radiotherapy and chemotherapy. As autoimmune diseases affect some antioxidant markers, some studies reported a significant difference in blood vitamin C levels in patients with autoimmune thyroid diseases such as Graves' disease. Despite many studies evaluating the effects of IV administration of vitamin C in mentioned diseases, there is a lack of evidence for oral consumption of vitamin C.

**Conclusions:**

To conclude, there is a lack of evidence, especially clinical trials, for the therapeutic effects of vitamin C on thyroid diseases; however, promising results were reported in some studies in the literature.

## Graphical abstract

Vitamin C is involved in the maintenance of several body functions, and its role has been shown in several organs and systems. Due to the lack of a comprehensive review regarding the links between vitamin C and the thyroid, in this article, we aimed to review the association of vitamin C and the thyroid in several diseases such as thyroid cancers, goitre, Graves' disease and hypo‐ and hyperthyroidism.

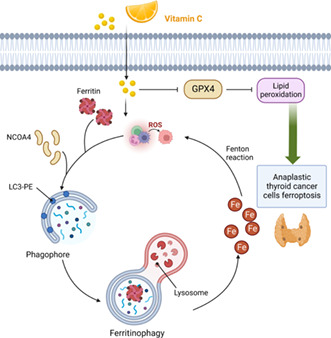

## 1 INTRODUCTION

Vitamin C is involved in the maintenance of several body functions, and its role has been shown in several organs and systems.^1^ Although its main form in the body is ascorbate, it acts as a co‐substrate for several enzymes and antioxidants.^2^, ^3^ Its role as an antioxidant is well known and it is well known as reactive oxygen species (ROS) scavenger in neurons.^4^, ^5^ Its concentration in body fluids and tissues depends on intestinal absorption, cellular transport and excretion.^6^ The thyroid gland, as one of the main sources of controlling metabolism and growth, plays an important role in body function, mainly through its main hormones, thyroxine and triiodothyronine.^7^ Several studies have assessed the levels of vitamin C in thyroid disorders or the effects of vitamin C on the absorption of thyroid medications. However, the effect of vitamin C on thyroid function and hormones is still unknown. Herein, by reviewing the available literature, we aimed to assess the association of vitamin C on the thyroid function gland, whether as a supplement or as a peripheral biomarker by comparing its levels among patients and controls. The search terms associated with our findings are shown in Table S1. The overall findings can be categorized into the following sections: (1) thyroid cancer, (2) goitre, (3) thyroid autoimmune disorders including Graves' disease and autoimmune thyroiditis, (4) role of vitamin C in levothyroxine absorption, (5) oxidative stress, vitamin C, and hypothyroidism, (6) vitamin C effects on oxidative stress of hyperthyroid patients, (7) benign thyroid disease and (8) thyroid lesions.

## 2 CANCER

A growing body of evidence documented the anti‐cancer potential of vitamin C,^8^, ^9^ and several pre‐clinical and clinical studies have confirmed this concept. The anti‐cancer effect of vitamin C was first introduced nearly 50 years ago by Pauling and Cameron.^10^ They showed in clinical studies that intravenous vitamin C (∼10 g daily) could increase the survival duration of patients with incurable cancer. Vitamin C was used as oral or intravenous administration and single or combined treatment; therefore, controversial findings were observed.

### 2.1 Anti‐cancer mechanism of vitamin C

Several mechanisms have been proposed for the cytotoxic effect of vitamin C in cancerous cells. Different studies have shown that vitamin C acts as a multi‐organ targeting agent and can play a role in epigenetic level, regulation of kinase activity, inhibition of epithelial‐to‐mesenchymal transition (EMT), immunoregulatory effect, increasing oxygen sensation, pro‐oxidant activity, etc.^11^, ^12^, ^13^, ^14^, ^15^ Pro‐oxidant activity is the main mechanism determined for the anti‐cancer activity of vitamin C and acts on redox imbalance in a dose‐dependent manner.^16^ The pro‐oxidant activity of vitamin C is mediated by inducing injury to deoxyribonucleic acid (DNA) molecules, inducing DNA mutation and genome instability.^17^ It is recognized that catalase activity was absent in cancerous cells that make them susceptible to oxidative stress regardless of the type of cancer.^18^ A high level of glucose transporter 1 (GLUT1) expression was observed in cancer cells that mediate uptake of vitamin C. Consequently, low levels of intracellular vitamin C mediate the reduction of antioxidants like nicotinamide adenine dinucleotide phosphate (NADPH) and superoxide dismutase (SOD) enzyme. Therefore, high doses of vitamin C can increase ROS levels in cancer cells and subsequently induce DNA, protein and lipid damage to cancerous cells. BRAF (v‐raf murine sarcoma viral oncogene homologue B1) mutation is the most frequent gene mutation responsible for the development of thyroid cancer and induces invasion of thyroid cancer. BRAF mutation showed their antitumour effects medicated by mitogen‐activated protein kinase/extracellular signal‐regulated protein kinase (MAPK/ERK) signalling.^19^ The anti‐cancer effect of vitamin C is shown to be medicated by directing BRAF mutation or even regardless of targeting BRAF mutation. Regarding targeting BRAF mutant thyroid cancer cells, it was shown that vitamin C plays its antitumour effect through inhibition of MAPK/ERK and PI3K/AKT pathway which was mediated by ROS‐dependent manner.^20^ Alongside, PLX4032 is an antitumour treatment option that inhibits BRAFV600 kinase. It was shown that vitamin C can play a role in enhancing the antitumour effect of PLX4032 by significantly increasing mitigation of MAPK/ERK as well as PI3K/AKT pathway.^21^ Moreover, another study showed that the induction of ROS production and decreasing antioxidant barrier of thyroid cancer cells by vitamin C selectively occurred in BRAF‐mutated cells.^22^ Redox homeostasis is responsible for several cellular mechanisms such as reaction to ROS and oxidation–reduction reaction. In this regard, it was shown that vitamin C has also an impact on redox haemostasis and subsequently on nicotinamide adenine dinucleotide (NAD) salvage mechanism and tricarboxylic acid (TCA) cycle which eventuate cell death.^22^

**FIGURE 1:**
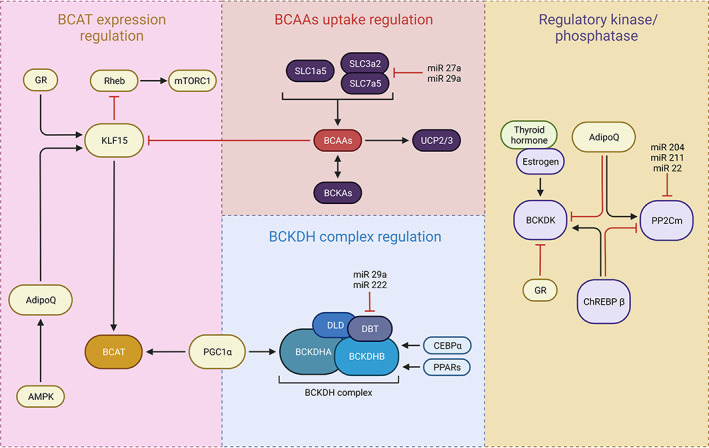
Transcriptional and hormonal regulators of BCAA catabolising enzymes and its association with thyroid hormone.

**FIGURE 2:**
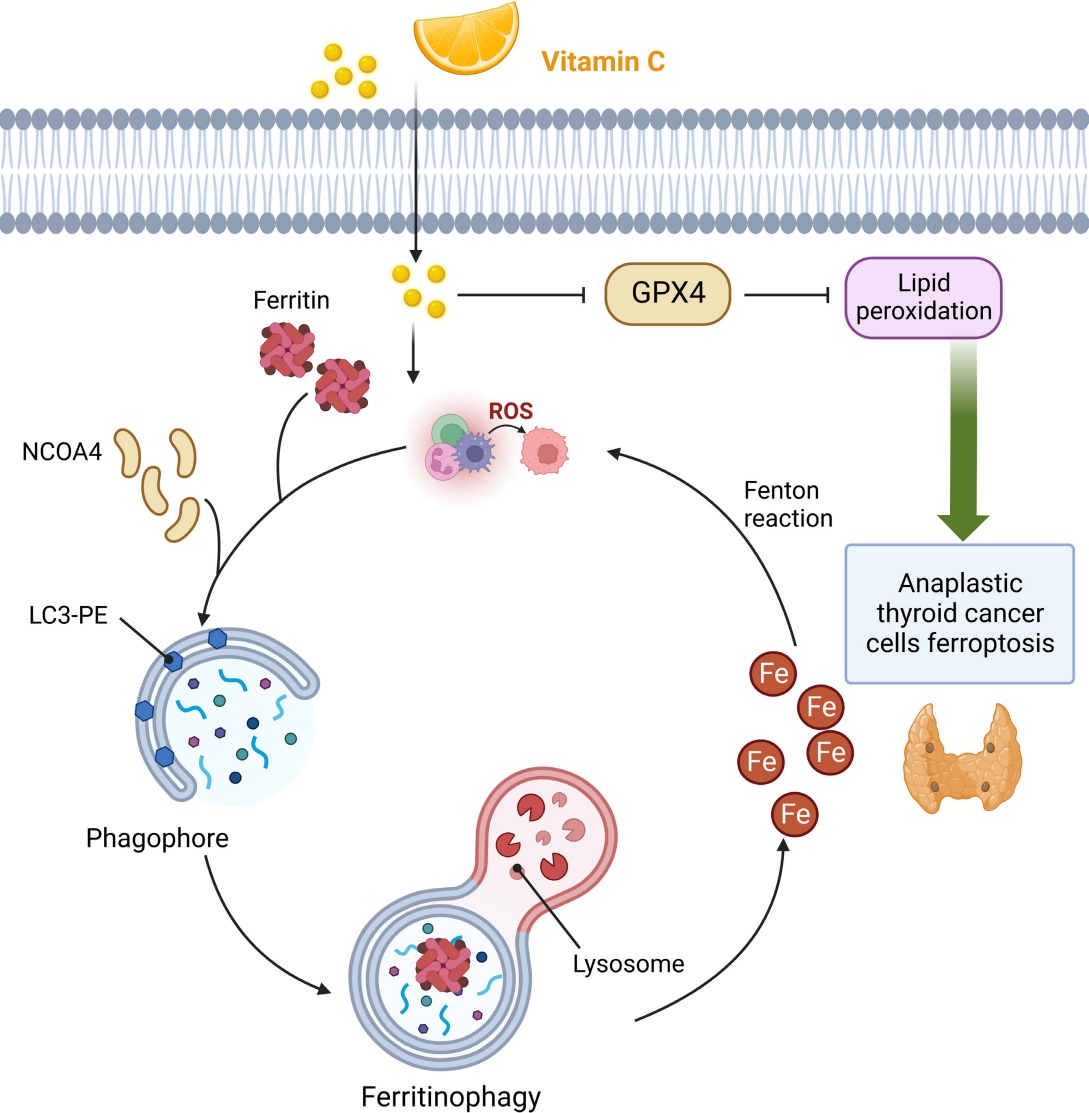
Anaplastic thyroid cancer cells mechanism in which vitamin C activates ferritinophagy to induce ferroptosis.

Vitamin C can also increase the apoptosis of cancerous cells by inhibiting B‐cell lymphoma‐2 (BCL‐2) expression and increasing the expression of BAX and caspase‐3 which results in cell apoptosis.^23^ Additionally, vitamin C can decrease hypoxia‐inducible factor‐1 (HIF‐1) which is essential for the endurance of cancerous cells in the hypoxia which increase the vulnerability of cancer cells to hypoxic condition.^24^ Moreover, vitamin C can bear therapeutic potential for cancer treatment through the activation of tumour suppressor genes like p53 and p21 which are associated with cell cycle arrest and inhibition of cancer proliferation.^23^ Furthermore, it was shown that vitamin C can play a role as a carcinostatic agent by inhibiting angiogenesis via decreasing the expression of angiogenesis‐related genes such as basic fibroblast growth factor (bFGF), vascular endothelial growth factor (VEGF) and matrix metallopeptidase 2 (MMP2).^25^ Anaplastic thyroid cancer (ATC) is a rare type of thyroid cancer that is associated with poor prognosis and there is no evidence of a definite treatment option for the management of ATC.^26^ It was shown that vitamin C can significantly decrease the proliferation and advancement of ATC cells by activation of ferroptosis and iron‐dependent lipid peroxidation. Vitamin C has been shown to have an effect on ferritinophagy that subsequently resulted in the degeneration of ferritin and discharge of free iron. Discharged free iron can subsequently stimulate ROS generation mediated by the Fenton reaction. The ROS reaction and lipid‐peroxidation induced by free iron can eventuate to ferroptosis and elimination of ATC cells.^27^ The effect of vitamin C is not limited to the impacts on cancerous cells. It was also documented that vitamin C can also stimulate the immune cells' reaction and can activate natural killer cells, T cells and monocyte that are responsible for the immune system in the fight against cancers.^28^, ^29^ The thyroid hormone plays a critical role in regulating branched‐chain amino acid (BCAA) metabolism by modulating the expression of branched‐chain aminotransferase (BCAT) and the activity of regulatory kinases and phosphatases involved in BCAA uptake. Figure 1 illustrates the key mechanisms involved in thyroid hormone‐mediated regulation of BCAA metabolism, highlighting the importance of understanding these pathways for developing targeted therapies for metabolic disorders and thyroid‐related diseases. Moreover, vitamin C plays a crucial role in regulating iron metabolism by promoting ferroportin‐mediated iron export and inhibiting hepcidin expression. Additionally, ferritinophagy, the autophagic degradation of ferritin, is an important mechanism for maintaining iron homeostasis and preventing oxidative stress caused by excess iron. Figure 2 illustrates the interplay between vitamin C, ferritinophagy and ROS in regulating iron metabolism.

### 2.2 Pre‐clinical studies

Pre‐clinical studies have shown that a millimolar range of vitamin C is required for destroying cancerous cells that are not reachable through oral administration, and intravenous infusion is the optimal route of administration.^30^, ^31^ Several pre‐clinical studies have investigated the effect of high doses of vitamin C on challenging tumours such as glioblastoma multiform (GBM), breast cancer, colorectal cancer, mesothelioma and pancreatic cancer and showed promising results on the anti‐cancer effect of vitamin C through hampering the tumour progression and tumour metastasis.^8^, ^16^ Applying high doses of vitamin C in KRAS gene mutation‐driven tumours has resulted in progression in pre‐clinical studies of vitamin C. It was shown that vitamin C directs mitochondrial membrane and metabolic components and reduces the levels of adenosine triphosphate (ATP) and GLUT1, which can also make KRAS mutant cells vulnerable to chemotherapy agents.^16^ Therefore, high doses of vitamin C opened up a new way to treat cancer and can be considered adjuvant therapy with other therapeutic options. Besides, vitamin C can also present as a protective treatment against the complications of other therapeutic agents meanwhile employed as a combination therapy.^25^

### 2.3 Clinical studies

In light of the anti‐cancer potential of vitamin C in cancer management, Phases I and II clinical trials reported favourable signatures of the safety and efficacy of vitamin C as combination therapy or even monotherapy in the treatment of various cancers.^9^, ^32^ Clinical studies of vitamin C monotherapy in terminal cancer showed that a high dose of vitamin C, up to 3 g/kg, has no considerable safety issues.^30^ In line with pre‐clinical studies, several clinical studies have documented that vitamin C can provide long‐term survival for patients with cancer, even with terminal or metastatic cancer.^16^ However, no clinical studies have investigated the efficacy of vitamin C monotherapy in patients with late‐stage cancer without preceding treatment. The key difference in the outcomes is thought to be related to the route of administration and pharmacokinetics of vitamin C.^31^ Hoffer et al.^33^ designed a clinical trial study on end‐stage cancer and showed that no patient had a response to ascorbic acid. Besides, it was also demonstrated that high‐dose oral administration of vitamin C is successful in reducing the risk of development of gastrointestinal cancers, cervix, colorectal and breast cancer.^34^, ^35^ Regarding oral administration of vitamin C, other studies revealed that daily intake of vitamin C is increased the survival of patients with breast cancer,^36^, ^37^ which implies that vitamin C has a clinically beneficial antitumour effect regardless of the route of administration. Even though high‐dose intravenous administration of vitamin C is associated with alternative medical agents in cancer treatment, there is a lack of sufficient evidence regarding the clinical efficacy of the antitumour effect of vitamin C.^38^ Therefore, it is essential to design further clinical studies addressing the clinical efficacy of the anticancer effect of vitamin C to set up appropriate evidence for the clinical efficacy of vitamin C in practical guidelines.

Cancers can influence patients' quality of life, and vitamin C is shown to positively affect pain relief and well‐being.^39^, ^40^ Moreover, vitamin deficiency is a common condition in patients with cancer, and anti‐neoplastic syndrome medications can also improve vitamin C deficiency and well‐being.^41^ Constitutional manifestations such as fatigue, depression, nausea, pain and loss of appetite are common in patients with cancer, and it was shown that intravenous administration of vitamin C reduces these complications and has palliative applications.^40^, ^42^ Altogether, high‐dose vitamin C was shown to prolong the survival duration of patients, better performances and less pain compared to the control patients without receiving vitamin C.^42^

### 2.4 Combination therapy

The effect of vitamin C as a combination therapy with other therapeutic options like chemotherapy, radiotherapy, targeted therapies and immunotherapy has also been studied.^43^ It was shown that vitamin C could enhance the efficacy of monotherapies agents like cisplatin,^23^ gemcitabine,^44^, ^45^, ^46^ sorafenib,^47^ PLX4032^21^ and 5‐fluorouracil^44^ in different types of cancers and provide significant tumour growth inhibition. However, 11 clinical studies on the combination therapy of ascorbic acid and arsenic trioxide (As_2_O_3_) were designed and documented that vitamin C has no anti‐cancer effect.^43^ Other studies have also investigated the efficacy of combination therapy of vitamin C with targeted therapies. Vitamin C can be used as a combination therapy with radiotherapy. However, it is well known that radiopharmaceutical agents like Iodine‐131 have mistaken toxicity on human lymphocytes through interruption of the double‐strand breaks (DSBs) repairing system and increasing the level of DSB that results in cell death and malignancy.^48^ Despite this, it was investigated that vitamins like vitamin E and vitamin C as antioxidant agents are game changers and can reduce the toxicity level of radiopharmaceuticals with a higher efficacy of vitamin C.^49^, ^50^ Clinical studies on the combination therapy of vitamin C with other therapeutic approaches demonstrated no obvious safety issues related to ascorbic acid. In two clinical trials of combination therapy of ascorbic acid and gemcitabine, the patients' toxicity was attributed to the gemcitabine not related to vitamin C.^45^, ^46^

Studies documented that vitamin C has a synergic effect with kinase inhibitors like sorafenib,^47^ cetuximab^13^ and gefitinib^12^ which can also have related to eliminating drug resistance to these agents. Moreover, few studies have investigated the combination therapy of vitamin C and immunotherapy. It was shown that high‐dose vitamin C could enhance the immunogenicity of effector T cells nor regulatory T cells and have synergic effects with immune checkpoint inhibitors that significantly increased the immunogenicity.^51^, ^52^ In addition to optimising cancer therapies, vitamin C can reduce the complains related of chemotherapy and radiotherapy, enhance the quality‐of‐life of patients with cancers and have protective effects on the glands.^41^, ^53^

### 2.5 Vitamin C in thyroid cancers

Studies have shown that vitamin C has potential as an anti‐cancer agent in the treatment of thyroid cancer. Vitamin C has been found to sensitize BRAF V600E thyroid cancer to PLX4032, a targeted therapy, by relieving the feedback activation of MAPK/ERK and PI3K/AKT pathways.^21^ Mechanistic studies have also revealed that vitamin C inhibits the MAPK/ERK and PI3K/AKT signalling pathways in BRAF wild‐type or mutant thyroid cancer cells.^20^ Vitamin C has also been found to inhibit the growth of papillary thyroid carcinoma (PTC) cells.^22^ In addition, high‐dose intravenous vitamin C has been shown to be a promising multi‐targeting anti‐cancer agent in eradicating tumour cells of various cancer types, including thyroid cancer. Vitamin C induces ferroptosis in anaplastic thyroid cancer cells, which suggests its potential as a therapeutic agent.^16^

Vitamin C inhibits the MAPK/ERK and PI3K/AKT signalling pathways in thyroid cancer cells through a ROS‐dependent mechanism.^16^, ^20^ The data from these studies demonstrate that vitamin C kills thyroid cancer cells by inhibiting these pathways via distinct mechanisms.^54^ In addition, vitamin C ROS dependently inhibits the activity of MAPK/ERK signalling via distinct mechanisms between ATP levels in BRAF mutant and wild‐type thyroid cancer cells. Overall, the exact mechanisms by which vitamin C inhibits these pathways in thyroid cancer cells are not fully understood and require further investigation.^20^

Several studies evaluated the role of vitamin C in thyroid cancer as a protective agent against cancer, a radioprotective agent and its therapeutic potential. Table 1 summarizes the main findings of these studies. Five studies used only patients with differentiated thyroid cancers (DTC),^50^, ^53^, ^55^, ^56^, ^57^ two used patients with BRAF V600 E mutation thyroid cancer,^20^, ^58^ one in patients with PTC^22^ and one in patients with APC.^27^ Other studies used patients with all types of thyroid cancers in their study. Cheng et al.^55^ found that vitamin C increased the uptake index and excretion of parotid glands in patients with DTC. In line with the previous study, Tong et al.^53^ found that Vitamin C improved the secretory function of the parotid gland in patients with DTC who underwent radioiodine therapy. In contrast, Liu et al.^59^ found no benefits in adding vitamin C as vitamin C had no effect on the salivary absorbed dose of radioiodine at any time after its administration in patients with thyroid cancer. Two studies found radioprotective effects in patients receiving radioiodine therapy.^56^, ^57^ Another study in patients with DTC found that vitamin C (especially in combination with amifostine) can reduce the side effects of radioiodine therapy.^50^ Two studies found a potential therapeutic role for vitamin C in thyroid cancer patients with BRAF V600 E mutation as vitamin C has antitumour effects and can kill cancer cells by MAPK/ERK and PI3K/AKT pathways. In a recent study by Wang et al.^27^ supplementation with vitamin C supplementation led to ferritinophagy, leading to the release of free iron and the released iron rejiggered ROS production resulting in ferroptosis of ATC cells. Davanzo et al.^60^ found protection against thyroid cancer in patients receiving vitamin C; however, O'Grady et al.^61^ found an increased risk of thyroid cancer in patients with higher vitamin C intake.

**TABLE 1:** Studies evaluating the association between vitamin C in patients with thyroid cancer.

References	Year	Type of thyroid cancer	Main findings
Cheng et al.^55^	2022	Differentiated thyroid cancer	Supragingival scaling with vitamin C resulted in an increase in the uptake index of the bilateral parotid glands and the excretion rate of the left parotid gland
Davanzo et al.^60^	1997	Thyroid carcinoma	Protection against thyroid cancer was observed in patients receiving vitamin C
Jafari et al.^56^	2018	Differentiated thyroid cancer	Vitamin C has radioprotective effects in thyroid cancer patients receiving radioiodine therapy
Li et al.^50^	2017	Differentiated thyroid cancer	Amifostine and vitamin C can reduce the side effects of radioiodine treatment. They have even better outcomes when used together
Liu et al.^59^	2021	Thyroid cancer	Vitamin C cannot affect the salivary absorbed dose of radioiodine at any time after its administration
O'Grady et al.^61^	2014	Thyroid cancer	Vitamin C had an increased risk of thyroid cancer (*p* < .01)
Rosario et al.^57^	2016	Differentiated thyroid cancer	Vitamin C, as an antioxidant, can have protective effects in ablation with I‐131
Su et al.^58^	2021	BRAF (V600E) thyroid cancer	Vitamin C has antitumour effects of PLX4032 in BRAF(MT) thyroid cancer cells by relieving the feedback activation of MAPK/ERK and PI3K/AKT pathway. A combination of PLX4032 and vitamin C can be a potential therapeutic approach to treat BRAF(MT) thyroid cancer.
Su et al.^20^	2019	BRAF thyroid cancer	Vitamin C can kill thyroid cancer cells by inhibiting MAPK/ERK and PI3K/AKT pathways via a ROS‐dependent mechanism. This shows that the pharmaceutical concentration of vitamin C has potential clinical use in thyroid cancer therapy.
Tong et al.^53^	2020	Differentiated thyroid cancer	Vitamin C can improve the secretory function of the parotid gland in DTC patients undergoing radioiodine therapy
Tronci et al.^22^	2021	Papillary thyroid carcinoma	Vitamin C has antitumoural activity by altering redox homeostasis. It can also induce ROS production and depletion of antioxidant defences in PTC cells with BRAFV600 mutation. However, this was not true for the cells characterized by RET/PTC rearrangements, indicating the idea that this compound exerts a selective effect in tumour cells with specific mutations
Wang et al.^27^	2021	Anaplastic thyroid cancer	Vitamin C supplementation led to ferritinophagy and subsequent degradation of ferritin, leading to the release of free iron. The released iron rejiggered ROS production which resulted in ferroptosis of ATC cells.

## 3 GOITRE

Goitre is an enlarged thyroid gland due to several causes including but not limited to autoimmune disease, iron deficiency or thyroid nodules.^62^ Surgery is not the only treatment plan for all patients; however, for symptomatic moderate to large goitre and failure of medical treatment, surgery is the choice. The roles of vitamin C in goitre were described in several ways. Özdem et al.^63^ studied the effects of propylthiouracil treatment on serum and plasma antioxidant activities in toxic multinodular goitre patients. In hyperthyroid patients, the level of plasma vitamin C was lower but they had notably higher activities of the erythrocyte antioxidant enzymes. Another study by Aliciguzel et al.^64^ investigated plasma, erythrocyte and serum antioxidant activities in both newly diagnosed untreated toxic multinodular goitre patients (*n* = 22) and age‐ and sex‐matched healthy controls (*n* = 15). By measuring plasma vitamin C levels in both cases and controls, plasma vitamin C levels were significantly lower in patients compared to controls. A study conducted by Özbas et al.^65^ in 13 patients with toxic multinodular goitre, compared the baseline antioxidant levels with 3 months after complete thyroidectomy. In all patients, antithyroid medications were used to maintain patients' euthyroid before surgery, and L‐thyroxine replacement therapy was used following surgery. No significant difference in levels of vitamin C was seen in patients before and 3 months after surgery, suggesting no thyroid effect on vitamin C levels in these patients.

## 4 THYROID AUTOIMMUNE DISORDERS

Vitamin C has been shown to be involved in cellular functions of both innate and adaptive immune systems. Its antioxidant effects as a cofactor for numerous biosynthetic and gene regulatory enzymes play important roles in several immune‐modulating ways. These include neutrophil migration to the infection site, phagocytosis enhancement and generation of oxidants and also microbial killing. In this section, we aim to investigate the role of vitamin C in two autoimmune disorders of the thyroid such as Graves' disease and autoimmune thyroiditis.

### 4.1 Graves' disease

Graves' disease, the most common cause of hyperthyroidism, is an autoimmune disease affecting the thyroid gland.^66^ Herein, we provided a comparison of vitamin C levels between patients and controls in addition to the role of vitamin C in patients presenting with Graves' disease. In a randomized clinical trial by Vrca et al.^67^ patients with newly diagnosed Graves' disease were randomized to get methimazole (control group) or methimazole in combination with an antioxidant supplementation with a fixed combination of beta‐carotene, selenium and vitamins C and E (intervention group). Although no significant changes were detected in low‐density lipoprotein cholesterol (LDL‐C) levels of controls, the LDL‐C concentration significantly increased in the intervention group (2.34–3.32 mmol/L after 1 month). However, high‐density lipoproteins cholesterol levels both increased in control and intervention groups following the treatment. Although the trial provided information about the comparison of LDL‐C concentrations in Graves' disease patients, no measurement of ascorbic acid levels was performed.

Another study by Londzin‐Olesik et al.^68^ in patients with Graves' disease and active thyroid‐associated orbitopathy, investigated the impact of thyroid hormone levels on selected antioxidant markers. Patients were divided into hyperthyroid and euthyroid based on thyroid hormone levels. In addition, 20 age‐ and sex‐matched controls were also involved. Vitamin C levels were significantly lower in hyperthyroid compared to healthy controls; however, this difference was insignificant compared to euthyroid patients. Authors suggested this decrease in vitamin C levels to excessive use and increasing demand in hyperthyroid patients with Graves' disease. In patients with Graves' disease and active orbitopathy, Londzin‐Olesik et al.^69^ found decreasing levels of vitamin C after systemic intravenous and oral methylprednisolone compared to 20 healthy age‐ and sex‐matched controls. In individuals with thyroid‐associated orbitopathy, methylprednisolone therapy is beneficial in lowering both clinical symptoms and oxidative stress, emphasising the role of corticosteroids in treating Graves' disease. To conclude, there is little evidence concerning plasma vitamin C, Graves' disease and Graves' disease therapies.

### 4.2 Autoimmune thyroiditis

In a study conducted by Taddei et al.^70^ the role of systemic inflammation on the pathogenesis of endothelial dysfunction was examined in subclinical hypothyroidism with autoimmune thyroiditis in 53 patients and 45 healthy controls. The effects of intrabrachial vitamin C administration on the response to acetylcholine for assessing the role of oxidative stress were measured. Regarding comparing the potential to vasodilation to acetylcholine, vitamin C infusion significantly changed the vasodilation (from 3.4 ± 0.2 to 25.4 ± 1.3 mL/min dl) compared to the saline infusion (from 3.4 ± 0.2 to 14.9 ± 1.8 mL/min dl) in patients with autoimmune thyroiditis, without modifying vasodilation to acetylcholine in controls. Although the mechanism is unknown, authors suggested that the mechanism is endothelial dysfunction by oxidative stress production by cyclooxygenase activity.^71^, ^72^, ^73^

Only one randomized controlled trial by Karimi et al.^74^ investigated the effects of vitamin C in patients with autoimmune thyroiditis on levels of antithyroid peroxidase antibody in serum specimens. Patients received either 500 mg of vitamin C/day or a placebo for 3 months. They found no significant difference in levels of thyroid‐stimulating hormone (TSH) and Tg–Ab between vitamin C and the control group (*p* > .05). However, thyroid peroxidase (TPO)‐Ab levels significantly decreased after treatment with vitamin C and remained constant in placebo, emphasising the antioxidant benefit of vitamin C on antibodies specific to the thyroid.

## 5 ROLE OF VITAMIN C IN LEVOTHYROXINE ABSORPTION

The effects of vitamin C on the absorption of levothyroxine in hypothyroid individuals have been investigated in the literature. High doses of levothyroxine may be needed in some patients, and hypoacidic conditions in the gastric environment could influence the drug's absorption, leading to unresponsiveness to the medication. The impact could be to the extent that, in some case reports, parenteral levothyroxine was administered without a clear reason for the underlying pathophysiological malabsorption mechanism.^75^, ^76^, ^77^ The study by Antúnez et al.^78^ investigated the effect of vitamin C administration in 28 hypothyroid patients requiring >1.70 μg/kg of levothyroxine but with normal TSH levels. The TSH level was measured before and after treatment with 1 g/day of vitamin C administered with levothyroxine, while the levothyroxine dose was constant, and it was found that the patients had statistically lower TSH levels after the intervention.

Similarly, the study by Jubiz et al.^79^ which assessed the effect of vitamin C on the concentration of TSH, T3 and T4 in hypothyroid cases with gastrointestinal abnormalities and elevated TSH levels, concluded that vitamin C can increase T3, T4 and subsequently reduce TSH. While there is no clear explanation of vitamin C's effect on levothyroxine malabsorption, there has been a suggestion that high gastric pH may interfere with levothyroxine absorption, and therefore, decreasing pH via vitamin C can enhance the drug's absorption.^80^ This idea could be supported by a recent systematic review which demonstrated an increase in TSH levels in concomitant use of levothyroxine and proton pump inhibitors (PPIs).^81^ However, this might cause problems in hypothyroid patients in need of PPI use, and increased gastric pH while consuming oral ascorbic acid with levothyroxine may have potential benefits in increasing the drug's absorption.

## 6 OXIDATIVE STRESS, VITAMIN C AND HYPOTHYROIDISM

The study by Erdamar et al. investigated the antioxidant status markers, including malondialdehyde (MDA), nitrite, vitamin E, vitamin A, b‐carotene, ascorbate and the activities of SOD, and myeloperoxidase (MPO), in both patients with hypothyroidism and hyperthyroidism.^82^ Ascorbic acid levels were determined in control patients, in addition to ones with Hashimoto's thyroiditis, at baseline and after 30 and 60 days of treatment with levothyroxine. There was no statistical difference in vitamin C levels between untreated hypothyroid patients and healthy control at baseline; however, treatment with levothyroxine in hypothyroid cases had a significant association with an increase in vitamin C levels. As vitamin C is among non‐enzymatic antioxidants,^83^, ^84^ it was concluded that reactive oxygen species were increased in hypothyroidism, suggesting the oxidative stress caused by this condition.

## 7 VITAMIN C EFFECTS ON OXIDATIVE STRESS OF HYPERTHYROID PATIENTS

The impact of vitamin C supplementation on oxidative stress was investigated in hyperthyroid patients treated with propylthiouracil in the study by Seven et al.^85^ In this analytical study, supplemental ascorbic acid of 1000 mg/day was given to hyperthyroid patients and healthy controls. Several oxidative factors were measured, and it was found that vitamin C consumption was statistically associated with an increased glutathione concentration and glutathione peroxidase activity, while it was related to the reduction in the glutathione reductase and Cu/Zn superoxide dismutase activities. These observations suggest relief in oxidative stress in this population. In another study conducted in Turkey, hyperthyroid patients were assessed and compared with healthy controls for antioxidant levels.^82^ The authors showed that there was no significant difference between hyperthyroid patients and healthy subjects. Additionally, as what was observed in hypothyroid cases, one may expect a significant change in oxidative agent levels after administrating hyperthyroidism medications. However, there was a non‐significant increase in ascorbate levels after the use of propylthiouracil. This was unlike what was expected and what was observed in hypothyroid patients in which significant changes were seen in metabolic and oxidative states. Overall, as there is heterogeneity in study designs, treatments and dosages, there is a need for further research on this topic which can enable us to conclude.

## 8 BENIGN THYROID DISEASE

Moncayo et al.^86^ investigated the role of vitamin C in benign thyroid diseases. Subjects were divided into three categories: (1) immunogenic thyroid disease (*n* = 112), (2) subacute thyroiditis (*n* = 29) and (3) normal thyroid (*n* = 273). Levels of vitamin C were higher in blood samples of patients with subacute thyroiditis compared to immunogenic thyroid disease (6.48 ± 3.12 μg/L vs. 6.10 ± 2.63 μg/L). In addition, both abnormal thyroids populations had higher concentrations of vitamin C in comparison with healthy controls (6.07 ± 2.94 μg/L in normal thyroid). In addition, they found that low levels of vitamin C affect Se action and metabolism in benign thyroid disease patients.

## 9 THYROID LESIONS

In a research by Jóźwiak et al.^87^ the association between the expression of HIF‐1α and HIF‐2α and levels of vitamin C in thyroid lesions was studied. Researchers used thyroid lesions from 106 nodular thyroid disease patients who underwent surgical resection. They found an inverse relation between tissue ascorbate level and HIF‐1α expression (*r* = −0.288, *p* = .025). In addition, no difference between vitamin C levels of thyroid lesions was detected. The findings of this study suggest that intracellular vitamin C levels in thyroid lesions are not different and extracellular accumulation of vitamin C changes in thyroid lesions.

## 10 CONCLUSION

Vitamin C, or L‐ascorbic acid, is an essential vitamin with antioxidant properties, which is essential for both preventing and treating different diseases. We intended to offer a thorough overview of all human studies investigating the various roles of vitamin C in the thyroid gland because vitamin C is an antioxidant and thyroid function may be impacted and may affect vitamin C levels. In this review, we covered different diseases such as Graves' disease, goitre, thyroid cancer and other causes of hyperthyroidism and hypothyroidism. Additionally, a study of vitamin C and other drugs like levothyroxine was conducted. Vitamin C may rectify anomalies in serum‐free T4, T3 and TSH concentrations in patients with hypothyroidism and gastrointestinal disease. In addition, vitamin C may have anti‐cancer properties in addition to its advantageous effects when combined with chemotherapy. A study revealed that in patients with hypothyroidism and gastrointestinal pathology, vitamin C could improve the abnormalities in serum‐free T4, T3 and TSH concentrations. To conclude, although some evidence suggests roles for vitamin C in thyroid diseases, further research with a higher sample size and more accurate research methodology is warranted.

## AUTHOR CONTRIBUTIONS

**Bahareh Farasati Far:** Conceptualization (equal); visualization (equal); writing – original draft (equal). **Amir Hossein Behnoush:** Writing – original draft (equal); writing – review and editing (equal). **Elina Ghondaghsaz:** Writing – original draft (equal). **Mohammad Amin Habibi:** Visualization (equal); writing – original draft (equal). **Amirmohammad Khalaji:** Conceptualization (lead); investigation (equal); supervision (lead); writing – original draft (equal); writing – review and editing (equal).

## FUNDING INFORMATION

None.

## CONFLICT OF INTEREST STATEMENT

The authors declare no conflict of interest.

## DATA AVAILABILTY STATEMENT

Not applicable.

## Supporting information


Table S1.

## References

[1] Figueroa‐Méndez R , Rivas‐Arancibia S . Vitamin C in health and disease: its role in the metabolism of cells and redox state in the brain. Front Physiol. 2015;6:397. 26779027 10.3389/fphys.2015.00397 PMC4688356

[2] Rumsey SC , Levine M . Absorption, transport, and disposition of ascorbic acid in humans. J Nutr Biochem. 1998;9(3):116‐130.

[3] Wells WW , Xu DP . Dehydroascorbate reduction. J Bioenerg Biomembr. 1994;26(4):369‐377. 7844111 10.1007/BF00762777

[4] Ebrahimzadeh‐Bideskan A‐R , Hami J , Alipour F , Haghir H , Fazel A‐R , Sadeghi A . Protective effects of ascorbic acid and garlic extract against lead‐induced apoptosis in developing rat hippocampus. Metab Brain Dis. 2016;31:1123‐1132. 27311610 10.1007/s11011-016-9837-7

[5] Harrison FE , May JM . Vitamin C function in the brain: vital role of the ascorbate transporter SVCT2. Free Radic Biol Med. 2009;46(6):719‐730. 19162177 10.1016/j.freeradbiomed.2008.12.018 PMC2649700

[6] Jacob RA , Sotoudeh G . Vitamin C function and status in chronic disease. Nutr Clin Care. 2002;5(2):66‐74. 12134712 10.1046/j.1523-5408.2002.00005.x

[7] Shahid MA , Ashraf MA , Sharma S . Physiology, Thyroid Hormone. StatPearls. Treasure Island (FL). StatPearls Publishing; 2022. 29763182

[8] Nakanishi K , Hiramoto K , Sato EF , Ooi K . High‐dose vitamin C administration inhibits the invasion and proliferation of melanoma cells in mice ovary. Biol Pharm Bull. 2021;44(1):75‐81. 33390553 10.1248/bpb.b20-00637

[9] Polireddy K , Dong R , Reed G , et al. High dose parenteral ascorbate inhibited pancreatic cancer growth and metastasis: mechanisms and a phase I/IIa study. Sci Rep. 2017;7(1):17188. 29215048 10.1038/s41598-017-17568-8 PMC5719364

[10] Cameron E , Pauling L . Supplemental ascorbate in the supportive treatment of cancer: prolongation of survival times in terminal human cancer. Proc Natl Acad Sci. 1976;73(10):3685‐3689. 1068480 10.1073/pnas.73.10.3685 PMC431183

[11] Hapke RY , Haake SM . Hypoxia‐induced epithelial to mesenchymal transition in cancer. Cancer Lett. 2020;487:10‐20. 32470488 10.1016/j.canlet.2020.05.012 PMC7336507

[12] Lee KE , Hahm E , Bae S , Kang JS , Lee WJ . The enhanced tumor inhibitory effects of gefitinib and L‐ascorbic acid combination therapy in non‐small cell lung cancer cells. Oncol Lett. 2017;14(1):276‐282. 28693165 10.3892/ol.2017.6109 PMC5494887

[13] Jung SA , Lee DH , Moon JH , et al. L‐ascorbic acid can abrogate SVCT‐2‐dependent cetuximab resistance mediated by mutant KRAS in human colon cancer cells. Free Radic Biol Med. 2016;95:200‐208. 27012422 10.1016/j.freeradbiomed.2016.03.009

[14] Zeng LH , Wang QM , Feng LY , et al. High‐dose vitamin C suppresses the invasion and metastasis of breast cancer cells via inhibiting epithelial‐mesenchymal transition. Onco Targets Ther. 2019;12:7405‐7413. 31571901 10.2147/OTT.S222702 PMC6753468

[15] Ang A , Pullar JM , Currie MJ , Vissers MCM . Vitamin C and immune cell function in inflammation and cancer. Biochem Soc Trans. 2018;46(5):1147‐1159. 30301842 10.1042/BST20180169 PMC6195639

[16] Böttger F , Vallés‐Martí A , Cahn L , Jimenez CR . High‐dose intravenous vitamin C, a promising multi‐targeting agent in the treatment of cancer. J Exp Clin Cancer Res. 2021;40(1):343. 34717701 10.1186/s13046-021-02134-y PMC8557029

[17] Reuter S , Gupta SC , Chaturvedi MM , Aggarwal BB . Oxidative stress, inflammation, and cancer: how are they linked? Free Radic Biol Med. 2010;49(11):1603‐1616. 20840865 10.1016/j.freeradbiomed.2010.09.006 PMC2990475

[18] Doskey CM , Buranasudja V , Wagner BA , et al. Tumor cells have decreased ability to metabolize H_2_O_2_: implications for pharmacological ascorbate in cancer therapy. Redox Biol. 2016;10:274‐284. 27833040 10.1016/j.redox.2016.10.010 PMC5106370

[19] Xing M . Molecular pathogenesis and mechanisms of thyroid cancer. Nat Rev Cancer. 2013;13(3):184‐199. 23429735 10.1038/nrc3431 PMC3791171

[20] Su X , Shen Z , Yang Q , et al. Vitamin C kills thyroid cancer cells through ROS‐dependent inhibition of MAPK/ERK and PI3K/AKT pathways via distinct mechanisms. Theranostics. 2019;9(15):4461‐4473. 31285773 10.7150/thno.35219 PMC6599666

[21] Su X , Li P , Han B , et al. Vitamin C sensitizes BRAFV600E thyroid cancer to PLX4032 via inhibiting the feedback activation of MAPK/ERK signal by PLX4032. J Exp Clin Cancer Res. 2021;40(1):1‐12. 33468157 10.1186/s13046-021-01831-y PMC7816401

[22] Tronci L , Serreli G , Piras C , et al. Vitamin C cytotoxicity and its effects in redox homeostasis and energetic metabolism in papillary thyroid carcinoma cell lines. Antioxidants. 2021;10(5):809. 34065197 10.3390/antiox10050809 PMC8161084

[23] Zhou J , Chen C , Chen X , Fei Y , Jiang L , Wang G . Vitamin C promotes apoptosis and cell cycle arrest in oral squamous cell carcinoma. Front Oncol. 2020;10:976. 32587830 10.3389/fonc.2020.00976 PMC7298137

[24] Fischer AP , Miles SL . Ascorbic acid, but not dehydroascorbic acid increases intracellular vitamin C content to decrease hypoxia inducible factor‐1 alpha activity and reduce malignant potential in human melanoma. Biomed Pharmacother. 2017;86:502‐513. 28012930 10.1016/j.biopha.2016.12.056

[25] Yeom C‐H , Lee G , Park J‐H , et al. High dose concentration administration of ascorbic acid inhibits tumor growth in BALB/C mice implanted with sarcoma 180 cancer cells via the restriction of angiogenesis. J Transl Med. 2009;7:70. 19671184 10.1186/1479-5876-7-70 PMC2732919

[26] Jannin A , Escande A , Al Ghuzlan A , et al. Anaplastic thyroid carcinoma: an update. Cancers. 2022;14(4):1061. 35205809 10.3390/cancers14041061 PMC8869821

[27] Wang X , Xu S , Zhang L , et al. Vitamin C induces ferroptosis in anaplastic thyroid cancer cells by ferritinophagy activation. Biochem Biophys Res Commun. 2021;551:46‐53. 33714759 10.1016/j.bbrc.2021.02.126

[28] Kim J‐E , Cho H‐S , Yang H‐S , et al. Depletion of ascorbic acid impairs NK cell activity against ovarian cancer in a mouse model. Immunobiology. 2012;217(9):873‐881. 22306178 10.1016/j.imbio.2011.12.010

[29] Perez‐Cruz I , Carcamo JM , Golde DW . Vitamin C inhibits FAS‐induced apoptosis in monocytes and U937 cells. Blood. 2003;102(1):336‐343. 12623840 10.1182/blood-2002-11-3559

[30] Stephenson CM , Levin RD , Spector T , Lis CG . Phase I clinical trial to evaluate the safety, tolerability, and pharmacokinetics of high‐dose intravenous ascorbic acid in patients with advanced cancer. Cancer Chemother Pharmacol. 2013;72(1):139‐146. 23670640 10.1007/s00280-013-2179-9 PMC3691494

[31] Padayatty SJ , Sun H , Wang Y , et al. Vitamin C pharmacokinetics: implications for oral and intravenous use. Ann Intern Med. 2004;140(7):533‐537. 15068981 10.7326/0003-4819-140-7-200404060-00010

[32] Chen Q , Espey MG , Sun AY , et al. Pharmacologic doses of ascorbate act as a prooxidant and decrease growth of aggressive tumor xenografts in mice. Proc Natl Acad Sci USA. 2008;105(32):11105‐11109. 18678913 10.1073/pnas.0804226105 PMC2516281

[33] Hoffer LJ , Robitaille L , Zakarian R , et al. High‐dose intravenous vitamin C combined with cytotoxic chemotherapy in patients with advanced cancer: a phase I‐II clinical trial. PLOS One. 2015;10(4):e0120228. 25848948 10.1371/journal.pone.0120228 PMC4388666

[34] Moertel CG , Fleming TR , Creagan ET , Rubin J , O'Connell MJ , Ames MM . High‐dose vitamin C versus placebo in the treatment of patients with advanced cancer who have had no prior chemotherapy: a randomized double‐blind comparison. N Engl J Med. 1985;312(3):137‐141. 3880867 10.1056/NEJM198501173120301

[35] Block G . Vitamin C and cancer prevention: the epidemiologic evidence. Am J Clin Nutr. 1991;53(1):270S‐282S. 1985398 10.1093/ajcn/53.1.270S

[36] Harris HR , Orsini N , Wolk A . Vitamin C and survival among women with breast cancer: a meta‐analysis. Eur J Cancer. 2014;50(7):1223‐1231. 24613622 10.1016/j.ejca.2014.02.013

[37] Harris HR , Bergkvist L , Wolk A . Vitamin C intake and breast cancer mortality in a cohort of Swedish women. Br J Cancer. 2013;109(1):257‐264. 23736027 10.1038/bjc.2013.269 PMC3708583

[38] Padayatty SJ , Sun AY , Chen Q , Espey MG , Drisko J , Levine M . Vitamin C: intravenous use by complementary and alternative medicine practitioners and adverse effects. PloS One. 2010;5(7):e11414. 20628650 10.1371/journal.pone.0011414 PMC2898816

[39] Carr AC , McCall C . The role of vitamin C in the treatment of pain: new insights. J Transl Med. 2017;15(1):77. 28410599 10.1186/s12967-017-1179-7 PMC5391567

[40] Mansoor F , Kumar S , Rai P , et al. Impact of intravenous vitamin C Administration in reducing severity of symptoms in breast cancer patients during treatment. Cureus. 2021;13(5):e14867. 34113504 10.7759/cureus.14867 PMC8177022

[41] Vollbracht C , Schneider B , Leendert V , Weiss G , Auerbach L , Beuth J . Intravenous vitamin C administration improves quality of life in breast cancer patients during chemo−/radiotherapy and aftercare: results of a retrospective, multicentre, epidemiological cohort study in Germany. In Vivo. 2011;25(6):983‐990. 22021693

[42] Günes‐Bayir A , Kiziltan HS . Palliative vitamin C application in patients with radiotherapy‐resistant bone metastases: a retrospective study. Nutr Cancer. 2015;67(6):921‐925. 26168394 10.1080/01635581.2015.1055366

[43] Nauman G , Gray JC , Parkinson R , Levine M , Paller CJ . Systematic review of intravenous ascorbate in cancer clinical trials. Antioxidants. 2018;7(7):89. 30002308 10.3390/antiox7070089 PMC6071214

[44] O'Leary BR , Houwen FK , Johnson CL , et al. Pharmacological ascorbate as an adjuvant for enhancing radiation‐chemotherapy responses in gastric adenocarcinoma. Radiat Res. 2018;189(5):456‐465. 29547353 10.1667/RR14978.1 PMC6117840

[45] Monti DA , Mitchell E , Bazzan AJ , et al. Phase I evaluation of intravenous ascorbic acid in combination with gemcitabine and erlotinib in patients with metastatic pancreatic cancer. PLoS One. 2012;7(1):e29794. 22272248 10.1371/journal.pone.0029794 PMC3260161

[46] Welsh JL , Wagner BA , van't Erve TJ , et al. Pharmacological ascorbate with gemcitabine for the control of metastatic and node‐positive pancreatic cancer (PACMAN): results from a phase I clinical trial. Cancer Chemother Pharmacol. 2013;71(3):765‐775. 23381814 10.1007/s00280-013-2070-8 PMC3587047

[47] Rouleau L , Antony AN , Bisetto S , et al. Synergistic effects of ascorbate and sorafenib in hepatocellular carcinoma: new insights into ascorbate cytotoxicity. Free Radic Biol Med. 2016;95:308‐322. 27036367 10.1016/j.freeradbiomed.2016.03.031 PMC4867251

[48] Scherthan H , Hieber L , Braselmann H , Meineke V , Zitzelsberger H . Accumulation of DSBs in gamma‐H2AX domains fuel chromosomal aberrations. Biochem Biophys Res Commun. 2008;371(4):694‐697. 18457664 10.1016/j.bbrc.2008.04.127

[49] Safaei M , Jafarpour S , Mohseni M , et al. Vitamins e and C prevent DNA double‐strand breaks in peripheral lymphocytes exposed to radiations from iodine‐131. Indian J Nucl Med. 2018;33(1):20‐24. 29430110 10.4103/ijnm.IJNM_89_17 PMC5798093

[50] Li J , Jianhua J , Liu J , Wu Z , Li S . The protection of salivary glands by amifostine and vitamin C in radioiodine treatment. J Nucl Med. 2017;58:960265.

[51] Luchtel RA , Bhagat T , Pradhan K , et al. High‐dose ascorbic acid synergizes with anti‐PD1 in a lymphoma mouse model. Proc Natl Acad Sci U S A. 2020;117(3):1666‐1677. 31911474 10.1073/pnas.1908158117 PMC6983418

[52] Magrì A , Germano G , Lorenzato A , et al. High‐dose vitamin C enhances cancer immunotherapy. Sci Transl Med. 2020;12(532):eaay8707. 32102933 10.1126/scitranslmed.aay8707

[53] Tong H , Luo L , Wu C , Wang H , Cheng Y , Wu Z . Effects of vitamin C and selenium on salivary glands in patients with DTC treated with I. J Nucl Med. 2020;61:960265.

[54] Jingtai Z , Linfei H , Yuyang Q , et al. Targeting Aurora‐a inhibits tumor progression and sensitizes thyroid carcinoma to Sorafenib by decreasing PFKFB3‐mediated glycolysis. Cell Death Dis. 2023;14(3):224. 36990998 10.1038/s41419-023-05709-z PMC10060208

[55] Cheng Y , Tong HM , Li XQ , et al. Effect of vitamin E and supragingival scaling on salivary gland function in patients with differentiated thyroid cancer treated with I‐131. Nucl Med Commun. 2022;43(9):995‐1003. 35950355 10.1097/MNM.0000000000001605

[56] Jafari E , Alavi M , Zal F . The evaluation of protective and mitigating effects of vitamin C against side effects induced by radioiodine therapy. Radiat Environ Biophys. 2018;57(3):233‐240. 29860661 10.1007/s00411-018-0744-7

[57] Rosario PW , Batista KCS , Calsolari MR . Radioiodine‐induced oxidative stress in patients with differentiated thyroid carcinoma and effect of supplementation with vitamins C and E and selenium (antioxidants). Arch Endocrinol Metab. 2016;60(4):328‐332. 26910631 10.1590/2359-3997000000128 PMC10118725

[58] Su X , Li P , Han B , et al. Vitamin C sensitizes BRAF(V600E) thyroid cancer to PLX4032 via inhibiting the feedback activation of MAPK/ERK signal by PLX4032. J Exp Clin Cancer Res. 2021;40(1):34. 33468157 10.1186/s13046-021-01831-y PMC7816401

[59] Liu B , Kuang A , Huang R , et al. Influence of vitamin C on salivary absorbed dose of 131I in thyroid cancer patients: a prospective, randomized, single‐blind, controlled trial. J Nucl Med. 2010;51(4):618‐623. 20237029 10.2967/jnumed.109.071449

[60] Davanzo B , Ron E , LaVecchia C , Franceschi S , Negri E , Ziegler R . Selected micronutrient intake and thyroid carcinoma risk. Cancer. 1997;79(11):2186‐2192. 9179066

[61] O'Grady TJ , Kitahara CM , DiRienzo AG , Gates MA . The association between selenium and other micronutrients and thyroid cancer incidence in the NIH‐AARP diet and health study. PLoS One. 2014;9(10):e110886. 25329812 10.1371/journal.pone.0110886 PMC4203851

[62] Hughes K , Eastman C . Goitre ‐ causes, investigation and management. Aust Fam Physician. 2012;41(8):572‐576. 23145396

[63] Ozdem S , Aliciguzel Y , Ozdem SS , Karayalcin U . Effects of propylthiouracil treatment on antioxidant activities in blood of toxic multinodular goiter patients. Pharmacology. 2000;61(1):31‐36. 10895078 10.1159/000028377

[64] Alicigüzel Y , Ozdem SN , Ozdem SS , et al. Erythrocyte, plasma, and serum antioxidant activities in untreated toxic multinodular goiter patients. Free Radic Biol Med. 2001;30(6):665‐670. 11295364 10.1016/s0891-5849(00)00509-8

[65] Özbaş S , Soyder A , Didem Kozaci L , Kavak T , Boylu S . The comparison of erythrocyte, plasma, and serum antioxidant activities in toxic multinodular goitre patients before and after surgery. Turk J Surg. 2008;24(4):189‐192.

[66] Davies TF , Andersen S , Latif R , et al. Graves' disease. Nat Rev Dis Primers. 2020;6(1):52. 32616746 10.1038/s41572-020-0184-y

[67] Vrca VB , Mayer L , Skreb F , Rahelić D , Marušić S . Antioxidant supplementation and serum lipids in patients with Graves' disease: effect on LDL‐cholesterol. Acta Pharm. 2012;62(1):115‐122. 22472454 10.2478/v10007-012-0005-2

[68] Londzin‐Olesik M , Kos‐Kudła B , Nowak A , Wielkoszyński T , Nowak M . The effect of thyroid hormone status on selected antioxidant parameters in patients with Graves' disease and active thyroid‐associated orbitopathy. Endokrynol Pol. 2020;71(5):418‐424. 32797475 10.5603/EP.a2020.0049

[69] Londzin‐Olesik M , Kos‐Kudla B , Karpe J , Nowak A , Nowak M . The effect of immunosuppression on selected antioxidant parameters in patients with Graves' disease with active thyroid‐associated orbitopathy. Exp Clin Endocrinol Diabetes. 2021;129(10):762‐769. 33157557 10.1055/a-1274-0998

[70] Taddei S , Caraccio N , Virdis A , et al. Low‐grade systemic inflammation causes endothelial dysfunction in patients with Hashimoto's thyroiditis. J Clin Endocrinol Metab. 2006;91(12):5076‐5082. 16968790 10.1210/jc.2006-1075

[71] Saeed SA , Ahmed S . Anti‐ischemic effects of nimesulide, a cyclooxygenase‐2 inhibitor on the ischemic model of rabbit induced by isoproterenol. Arch Pharm Res. 2006;29(11):977‐983. 17146966 10.1007/BF02969281

[72] O'Banion MK . Cyclooxygenase‐2: molecular biology, pharmacology, and neurobiology. Crit Rev Neurobiol. 1999;13(1):45‐82. 10223523 10.1615/critrevneurobiol.v13.i1.30

[73] Katusic ZS , Vanhoutte PM . Superoxide anion is an endothelium‐derived contracting factor. Am J Physiol. 1989;257(1 Pt 2):H33‐H37. 2546450 10.1152/ajpheart.1989.257.1.H33

[74] Karimi F , Omrani GR . Effects of selenium and vitamin C on the serum level of antithyroid peroxidase antibody in patients with autoimmune thyroiditis. J Endocrinol Invest. 2019;42(4):481‐487. 30182359 10.1007/s40618-018-0944-7

[75] Tönjes A , Karger S , Koch CA , et al. Impaired enteral levothyroxine absorption in hypothyroidism refractory to oral therapy after thyroid ablation for papillary thyroid cancer: case report and kinetic studies. Thyroid. 2006;16(10):1047‐1051. 17042692 10.1089/thy.2006.16.1047

[76] Jauk B , Mikosch P , Gallowitsch HJ , et al. Unusual malabsorption of levothyroxine. Thyroid. 2000;10(1):93‐95. 10691319 10.1089/thy.2000.10.93

[77] Nagaoka T , Miyakoshi H , Takamura T , et al. A case of refractory hypothyroidism requiring daily intravenous thyroxine. J Int Med Res. 2002;30(4):463‐465. 12235934 10.1177/147323000203000418

[78] Antúnez P , Licht S . Vitamin C improves the apparent absorption of levothyroxine in a subset of patients receiving this hormone for primary hypothyroidism. Rev Argent Endocrinol Metab. 2011;48(1):16‐24.

[79] Jubiz W , Ramirez M . Effect of vitamin C on the absorption of levothyroxine in patients with hypothyroidism and gastritis. J Clin Endocrinol Metab. 2014;99(6):E1031‐E1034. 24601693 10.1210/jc.2013-4360

[80] Centanni M , Gargano L , Canettieri G , et al. Thyroxine in goiter, helicobacter pylori infection, and chronic gastritis. N Engl J Med. 2006;354(17):1787‐1795. 16641395 10.1056/NEJMoa043903

[81] Guzman‐Prado Y , Vita R , Samson O . Concomitant use of levothyroxine and proton pump inhibitors in patients with primary hypothyroidism: a systematic review. J Gen Intern Med. 2021;36(6):1726‐1733. 33469743 10.1007/s11606-020-06403-y PMC8175524

[82] Erdamar H , Demirci H , Yaman H , et al. The effect of hypothyroidism, hyperthyroidism, and their treatment on parameters of oxidative stress and antioxidant status. Clin Chem Lab Med. 2008;46(7):1004‐1010. 18605962 10.1515/CCLM.2008.183

[83] Frei B . Molecular and biological mechanisms of antioxidant action. FASEB J. 1999;13(9):963‐964. 10336879 10.1096/fasebj.13.9.963

[84] Freeman BA , Crapo JD . Biology of disease: free radicals and tissue injury. Lab Invest. 1982;47(5):412‐426. 6290784

[85] Seven A , Taşan E , İnci F , Hatemi H , Burçak G . Biochemical evaluation of oxidative stress in propylthiouracil treated hyperthyroid patients. Effects of Vitamin C Supplementation. Clin Chem Lab Med. 1998;36:767‐770. 9853803 10.1515/CCLM.1998.136

[86] Moncayo R , Kroiss A , Oberwinkler M , et al. The role of selenium, vitamin C, and zinc in benign thyroid diseases and of selenium in malignant thyroid diseases: low selenium levels are found in subacute and silent thyroiditis and in papillary and follicular carcinoma. BMC Endocr Disord. 2008;8:2. 18221503 10.1186/1472-6823-8-2 PMC2266752

[87] Jóźwiak P , Krześlak A , Wieczorek M , Lipińska A . Effect of glucose on GLUT1‐dependent intracellular ascorbate accumulation and viability of thyroid cancer cells. Nutr Cancer. 2015;67(8):1333‐1341. 26381034 10.1080/01635581.2015.1078823

