# Supplementary material for: The interplay between vitamin C and thyroid
Source: Endocrinol Diabetes Metab. 2023 May 29;6(4):e432. doi: 10.1002/edm2.432 (PMC10335618; doi:10.1002/edm2.432)
Supplement: Supplementary file 1 — Table S1. [file EDM2-6-e432-s001.docx]

Supplementary Material

***Supplementary Table 1.*** Search details

| **Query** | | **Results**  **(10 September 2022)** |
| --- | --- | --- |
| ***PubMed*** | | |
| #1 | (“Ascorbic Acid”[Mesh] OR “Ascorbic Acid”[tiab] OR “Acid, Ascorbic”[tiab] OR “L-Ascorbic Acid”[tiab] OR “Acid, L-Ascorbic”[tiab] OR “L Ascorbic Acid”[tiab] OR ”Vitamin C”[tiab] OR “Hybrin”[tiab] OR “Magnorbin”[tiab] OR “Sodium Ascorbate”[tiab] OR “Ascorbate, Sodium”[tiab] OR “Ascorbic Acid, Monosodium Salt”[tiab] OR “Ferrous Ascorbate”[tiab] OR “Ascorbate, Ferrous”[tiab] OR “Magnesium Ascorbate”[tiab] OR “Ascorbate, Magnesium”[tiab] OR “Magnesium di-L-Ascorbate”[tiab] OR “Magnesium di L Ascorbate”[tiab] OR “di-L-Ascorbate, Magnesium”[tiab] OR “Magnesium Ascorbicum”[tiab]) | 71,557 |
| #2 | ("Thyroid Gland"[Mesh] OR "Thyroid Gland"[tiab] OR “Thyroid*”[tiab] OR "Thyroid Hormones"[Mesh] OR "Thyroid Hormon*"[tiab] OR "Thyroxine"[Mesh] OR "Thyroxine"[tiab] OR “3,5,3',5'-Tetraiodothyronine”[tiab] OR “T4 Thyroid Hormone”[tiab] OR “Thyroid Hormone, T4”[tiab] OR “Synthrox”[tiab] OR “Levothyroxine Sodium”[tiab] OR “Sodium Levothyroxine”[tiab] OR “Thyrax”[tiab] OR “Tiroidine”[tiab] OR “Tiroxina Leo”[tiab] OR “Unithroid”[tiab] OR “Eferox”[tiab] OR “Eltroxin”[tiab] OR “Thevier”[tiab] OR “Eltroxine”[tiab] OR “Euthyrox”[tiab] OR “Eutirox”[tiab] OR “L-Thyrox”[tiab] OR “L Thyrox”[tiab] OR “L-Thyroxin beta”[tiab] OR “L Thyroxin beta”[tiab] OR “L-Thyroxin Henning”[tiab] OR “L Thyroxin Henning”[tiab] OR “Levothyroxine”[tiab] OR “O-(4-Hydroxy-3,5-diiodophenyl) 3,5-diiodo-L-tyrosine”[tiab] OR “L-Thyroxine”[tiab] OR “L Thyroxine”[tiab] OR “L-3,5,3',5'-Tetraiodothyronine”[tiab] OR “Levoxine”[tiab] OR “Levoxyl”[tiab] OR “Lévothyrox”[tiab] OR “L-Thyroxine Roche”[tiab] OR “L Thyroxine Roche”[tiab] OR “Levo-T”[tiab] OR “Levo T”[tiab] OR “Levothroid”[tiab] OR “Novothyral”[tiab] OR “Berlthyrox”[tiab] OR “Dexnon”[tiab] OR “Novothyrox”[tiab] OR “Oroxine”[tiab] OR “Synthroid”[tiab] OR “Levothyroxin Deladande”[tiab] OR “Levothyroxin Delalande”[tiab] OR “Levothyroid”[tiab] OR "Triiodothyronine"[Mesh] OR "Triiodothyronine"[tiab] OR “T3 Thyroid Hormone”[tiab] OR “Thyroid Hormone, T3”[tiab] OR “Liothyronine”[tiab] OR “3,3',5-Triiodothyronine”[tiab] OR “Liothyronine Sodium”[tiab] OR “Cytomel”[tiab]) | 266,089 |
| **#3** | **#1 AND #2** | 457 |
| ***SCOPUS*** | | |
| #1 | ((TITLE-ABS-KEY(“Acid”) AND TITLE-ABS-KEY(”Ascorbic”)) OR (TITLE-ABS-KEY(“L-Ascorbic”) AND TITLE-ABS-KEY(”Acid”)) OR (TITLE-ABS-KEY(“L Ascorbic”) AND TITLE-ABS-KEY(”Acid”)) OR TITLE-ABS-KEY(“Vitamin C”) OR TITLE-ABS-KEY(“Hybrin“) OR TITLE-ABS-KEY(“Magnorbin“) OR TITLE-ABS-KEY(”Ascorbate”) OR TITLE-ABS-KEY(“Magnesium di-L-Ascorbate“) OR TITLE-ABS-KEY(“di-L-Ascorbate”) OR TITLE-ABS-KEY(“Magnesium Ascorbicum”)) | 185,605 |
| #2 | (TITLE-ABS-KEY(“Thyroid*”) OR TITLE-ABS-KEY("Thyroxine") OR TITLE-ABS-KEY(“3,5,3',5'-Tetraiodothyronine”) OR TITLE-ABS-KEY(“T4 Thyroid Hormone”) OR TITLE-ABS-KEY(“Synthrox”) OR TITLE-ABS-KEY(“Levothyroxine”) OR TITLE-ABS-KEY(“Thyrax”) OR TITLE-ABS-KEY(“Tiroidine”) OR TITLE-ABS-KEY(“Tiroxina Leo”) OR TITLE-ABS-KEY(“Unithroid”) OR TITLE-ABS-KEY(“Eferox”) OR TITLE-ABS-KEY(“Eltroxin”) OR TITLE-ABS-KEY(“Thevier”) OR TITLE-ABS-KEY(“Eltroxine”) OR TITLE-ABS-KEY(“Euthyrox”) OR TITLE-ABS-KEY(“Eutirox”) OR TITLE-ABS-KEY(“L-Thyrox”) OR TITLE-ABS-KEY(“L Thyrox”) OR TITLE-ABS-KEY(“L-Thyroxin”) OR TITLE-ABS-KEY(“O-(4-Hydroxy-3,5-diiodophenyl) 3,5-diiodo-L-tyrosine”) OR TITLE-ABS-KEY(“L-Thyroxine”) OR TITLE-ABS-KEY(“L-3,5,3',5'-Tetraiodothyronine”) OR TITLE-ABS-KEY(“Levoxine”) OR TITLE-ABS-KEY(“Levoxyl”) OR TITLE-ABS-KEY(“Lévothyrox”) OR TITLE-ABS-KEY(“Levo-T”) OR TITLE-ABS-KEY(“Levo T”) OR TITLE-ABS-KEY(“Levothroid”) OR TITLE-ABS-KEY(“Novothyral”) OR TITLE-ABS-KEY(“Berlthyrox”) OR TITLE-ABS-KEY(“Dexnon”) OR TITLE-ABS-KEY(“Novothyrox”) OR TITLE-ABS-KEY(“Oroxine”) OR TITLE-ABS-KEY(“Synthroid”) OR TITLE-ABS-KEY(“Levothyroid”) OR TITLE-ABS-KEY("Triiodothyronine”) OR TITLE-ABS-KEY(“T3 Thyroid Hormone”) OR TITLE-ABS-KEY(“Liothyronine”) OR TITLE-ABS-KEY(“3,3',5-Triiodothyronine”) OR TITLE-ABS-KEY(“Liothyronine Sodium”) OR TITLE-ABS-KEY(“Cytomel”)) | 366,947 |
| **#3** | **#1 AND #2** | 1,231 |
| ***Embase*** | | |
| #1 | ((“Acid” AND ”Ascorbic”) OR (“L-Ascorbic” AND ”Acid”) OR (“L Ascorbic” AND ”Acid”) OR “Vitamin C” OR “Hybrin“ OR “Magnorbin“ OR ”Ascorbate” OR “Magnesium di-L-Ascorbate“ OR “di-L-Ascorbate” OR “Magnesium Ascorbicum”) | 133,763 |
| #2 | ('thyroid*' OR 'thyroxine' OR 't4 thyroid hormone' OR 'synthrox' OR 'levothyroxine' OR 'thyrax' OR 'tiroidine' OR 'tiroxina leo' OR 'unithroid' OR 'eferox' OR 'eltroxin' OR 'thevier' OR 'eltroxine' OR 'euthyrox' OR 'eutirox' OR 'l-thyrox' OR 'l thyrox' OR 'l-thyroxin' OR 'o-(4-hydroxy-3,5-diiodophenyl) 3,5-diiodo-l-tyrosine' OR 'l-thyroxine' OR 'tetraiodothyronine' OR 'levoxine' OR 'levoxyl' OR 'lévothyrox' OR 'levo-t' OR 'levo t' OR 'levothroid' OR 'novothyral' OR 'berlthyrox' OR 'dexnon' OR 'novothyrox' OR 'oroxine' OR 'synthroid' OR 'levothyroid' OR 't3 thyroid hormone' OR 'liothyronine' OR 'triiodothyronine' OR 'liothyronine sodium' OR 'cytomel') | 398,805 |
| **#3** | **#1 AND #2** | 1,184 |
| ***Web of Science*** | | |
| #1 | ((TS=“Acid” AND TS=”Ascorbic”) OR (TS=“L-Ascorbic” AND TS=”Acid”) OR (TS=“L Ascorbic” AND TS=”Acid”) OR TS=“Vitamin C” OR TS=“Hybrin“ OR TS=“Magnorbin“ OR TS=”Ascorbate” OR TS=“Magnesium di-L-Ascorbate“ OR TS=“di-L-Ascorbate” OR TS=“Magnesium Ascorbicum”) | 123,991 |
| #2 | (TS=“Thyroid*” OR TS="Thyroxine" OR TS=“3,5,3',5'-Tetraiodothyronine” OR TS=“T4 Thyroid Hormone” OR TS=“Synthrox” OR TS=“Levothyroxine” OR TS=“Thyrax” OR TS=“Tiroidine” OR TS=“Tiroxina Leo” OR TS=“Unithroid” OR TS=“Eferox” OR TS=“Eltroxin” OR TS=“Thevier” OR TS=“Eltroxine” OR TS=“Euthyrox” OR TS=“Eutirox” OR TS=“L-Thyrox” OR TS=“L Thyrox” OR TS=“L-Thyroxin” OR TS=“O-(4-Hydroxy-3,5-diiodophenyl) 3,5-diiodo-L-tyrosine” OR TS=“L-Thyroxine” OR TS=“L-3,5,3',5'-Tetraiodothyronine” OR TS=“Levoxine” OR TS=“Levoxyl” OR TS=“Lévothyrox” OR TS=“Levo-T” OR TS=“Levo T” OR TS=“Levothroid” OR TS=“Novothyral” OR TS=“Berlthyrox” OR TS=“Dexnon” OR TS=“Novothyrox” OR TS=“Oroxine” OR TS=“Synthroid” OR TS=“Levothyroid” OR TS="Triiodothyronine" OR TS=“T3 Thyroid Hormone” OR TS=“Liothyronine” OR TS=“3,3',5-Triiodothyronine” OR TS=“Liothyronine Sodium” OR TS=“Cytomel”) | 215,270 |
| **#3** | **#1 AND #2** | 348 |
| ***Total*** | | **3,220** |
| ***Total without duplicates*** | | **1,839** |
